# Two-dimensional analytical description of the plasma potential in a magnetron discharge

**DOI:** 10.1038/s41598-023-42949-7

**Published:** 2023-09-23

**Authors:** Claudiu Costin, Tiberiu M. Minea

**Affiliations:** 1 Faculty of Physics, Iasi Plasma Advanced Research Center (IPARC), Alexandru Ioan Cuza University of Iasi, 700506 Iasi, Romania; 2 Laboratoire de Physique des Gaz et des Plasmas, Université Paris-Saclay, CNRS, 91405 Orsay, France

**Keywords:** Magnetically confined plasmas, Information theory and computation

## Abstract

Simple analytical formulas are proposed to describe the plasma potential in a steady-state magnetron discharge, based on the results of various experiments and numerical simulations reported in the literature. The description is two-dimensional (2D), covering two main regions, the cathode sheath and the ionization region, both contributing to electron energization. A parabolic potential in the axial direction governs the cathode sheath. The thickness of the cathode sheath is obtained from the 1D collisionless Child–Langmuir law. A parabolic or linear potential in the axial direction characterizes the ionization region. The local ion current density to the cathode, estimated from the target erosion profile, sets the radial dependence of the potential. The proposed formulas use a set of input parameters that can be experimentally obtained. The analytical description captures all characteristics of the highly inhomogeneous plasma potential of a steady-state magnetron discharge operated in a reduced magnetic field *B*_*RT*_/*p* lower than 0.1 T/Pa, as revealed by the comparison to self-consistent 2D numerical simulations.

## Introduction

Magnetron sputtering is a leading technology for thin film deposition and coatings^1^. A complex magnetic field structure confines the plasma electrons in front of the cathode. Hence, the discharge operates at low pressure (~ 1 Pa) with a high-density plasma (10^16^–20^21^ m^−3^), depending on the excitation mode. The heavy species are gaseous and sputtered particles, neutral or ionized. The ions are practically not magnetized, having the Larmor radius much larger than the specific dimension of the magnetic trap. The low pressure facilitates the transport of the sputtered neutrals to the substrate, while the electric field controls the transport of the sputtered ions within the discharge.

The magnetron discharge volume is commonly divided into three regions: the cathode sheath (CS), the ionization region (IR), and the diffusion region (DR)^2^. The name of the ‘ionization region’ originates in the ionization region model (IRM) developed for the study of a high-power impulse magnetron sputtering (HiPIMS) system^3^. The IR corresponds to the negative glow, where the magnetic structure efficiently traps the electrons, and most ionizations occur. It is also known as the pre-sheath^4^. The diffusion region, or the bulk plasma^2,4^, connects the IR to the anode or substrate. The cathode sheath regulates the discharge since the electric field in this region is responsible for ion acceleration towards the cathode (target), sputtering, and a significant part of the ionization processes via secondary electron acceleration.

For the study of a magnetron discharge, the knowledge of the potential map in all three regions is essential for a large number of investigations, such as the transport of electrons^5,6^ and ions^7,8^ within the discharge, the study of the secondary electron emission process^9^, the formation and evolution of rotating azimuthal structures (named spokes)^10^, etc. Also, it can provide better input for volume-averaged (kinetic^11^, collisional-radiative^12^, phenomenological^13^, etc.) or zonal-averaged^14^ models.

The importance of the CS for the magnetron discharge operation was widely discussed in the literature^15–17^. Nevertheless, potential measurements in the CS are rather limited. While the DR and the anode side of the IR are accessible for probe measurements, whether in direct-current (DC)^4,18,19^ or HiPIMS mode^20–22^, the measurements in the CS and the cathode side of the IR are problematic due to high strength and gradients of the magnetic field and due to large plasma perturbations induced by the probe in this region^21^. Optical measurements seem more appropriate, the CS thickness being measured by optical emission spectroscopy^23^ or laser-induced fluorescence^15,24^. However, such measurements estimate only the minimum CS thickness corresponding to the racetrack (RT, the sputtered region of the target), while the CS thickness is non-uniform across the cathode. There was also an attempt to measure the electric field in the CS by laser-induced fluorescence^24^, but at very high pressure (850 mTorr ≈ 113 Pa), far from the typical operation of the magnetron discharge.

On the other hand, self-consistent numerical simulations like particle-in-cell (PIC)^25–32^ provide better insights into the spatial description of the local potential in front of the magnetron cathode. However, this modeling approach has a major drawback—the significant computational time—that makes the use of PIC inconvenient when a parametric study with multiple variables is required. To overcome these limitations, we propose simple analytical formulas giving a fast and educated estimate of the 2D potential map in front of a magnetron target. The formulas were obtained after analyzing a wide range of numerical and experimental results reported in the literature^4,22,24–28,30–33^. The analytical description of the potential can be further used in subsequent calculations (as in^5–14^) or interpreting experimental results (when the knowledge of the local electric field is required). It is suitable for the steady-state operation of magnetron discharges, although it was found to also comply with particular moments of transitory operation modes. Therefore, besides DC cases, references to transitory discharges are also indicated throughout the manuscript. However, a thorough description of transitory discharges is beyond the purpose of this paper. The analytical formulas use a limited number of input parameters, as further detailed.

## Analytical formulas for the plasma potential

The proposed analytical formulas give the two-dimensional map of the potential inside the cathode sheath and the ionization region of a steady-state magnetron discharge since the plasma potential in the diffusion region, *V*_*p*_, is known to be almost flat^18,19,22,30,32^.

Let us consider the common case in the literature—a planar magnetron with cylindrical symmetry. For this reason, the problem is formulated in (*r*,*z*) coordinates. The center of the cathode is positioned at (*r*,*z*) = (0,0), with *r* the radial coordinate parallel to the target and *z* the axial coordinate. The analysis can be easily adapted to Cartesian coordinates by replacing *r* with *x*, where *x* is the axis corresponding to the width of a rectangular target (the transverse direction). The anode facing the cathode is grounded, and the cathode is biased at −*U*_*d*_.

The analytical description of the potential is based on six input parameters: the discharge voltage *U*_*d*_, the discharge current *I*_*d*_, the target erosion profile χ(*r*), the limit of the ionization region towards the anode (or substrate) *Z*_*IR*_, the intercept at the cathode (*z* = 0) of the voltage drop law in the ionization region *U*_0_, and the plasma potential in the diffusion region *V*_*p*_. The first three parameters can be easily measured. The latter three can be estimated from probe measurements^4,18,20–22^, from models like the IRM (global model)^34^, or introduced as parametric variables in the proposed formulas.

**Figure 1:**
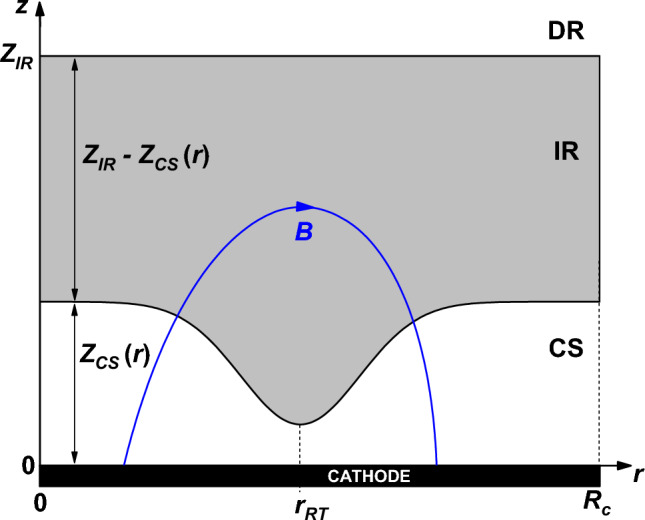
Schematic representation of the discharge regions in front of the magnetron cathode. A magnetic field line is shown in blue. The maximum of the target sputtering (racetrack) corresponds to *r*_*RT*_, where the magnetic field line is parallel to the cathode.

Concerning the axial electric field in the ionization region, the literature reports either constant^25,26,30^ or linear axial fields^4,27,31^, or even both^22,32^, depending on the discharge operation conditions. The limit of the IR towards the anode is assumed parallel to the cathode (*Z*_*IR*_ is independent of the radial position). In contrast, the limit of the cathode sheath, *Z*_*CS*_, highly varies with the radial position (as shown later), mirroring the curvature of the magnetic field lines (Fig. 1). Thus, the general expression for the potential axial dependency inside the ionization region can be written as:

1a $${V}_{IR}\left(z\right)=-{U}_{0}{\left(\frac{{Z}_{IR}-z}{{Z}_{IR}}\right)}^{n}+{V}_{p},$$

which corresponds to an electric field:

1b $${E}_{IR}\left(z\right)=-n\frac{{U}_{0}}{{Z}_{IR}}{\left(\frac{{Z}_{IR}-z}{{Z}_{IR}}\right)}^{n-1}.$$

The electric field in the IR is constant for *n* = 1 and linear for *n* = 2. Equations (1a) and (1b) are independent of the radial position because the input parameters *U*_0_, *V*_*p,*_ and *Z*_*IR*_ are assumed independent of *r*. However, the voltage drop over the IR, *U*_*IR*_, depends on *r* because the IR extension in the axial direction changes with the width of the CS (Fig. 1). According to Eq. (1a), the voltage drop over the IR writes:

2 $${U}_{IR}\left(r\right)={V}_{p}-{V}_{IR}\left({Z}_{CS}\right)={U}_{0}{\left(\frac{{Z}_{IR}-{Z}_{CS}\left(r\right)}{{Z}_{IR}}\right)}^{n},$$

being smaller than *U*_*0*_, regardless of the radial position.

**Figure 2:**
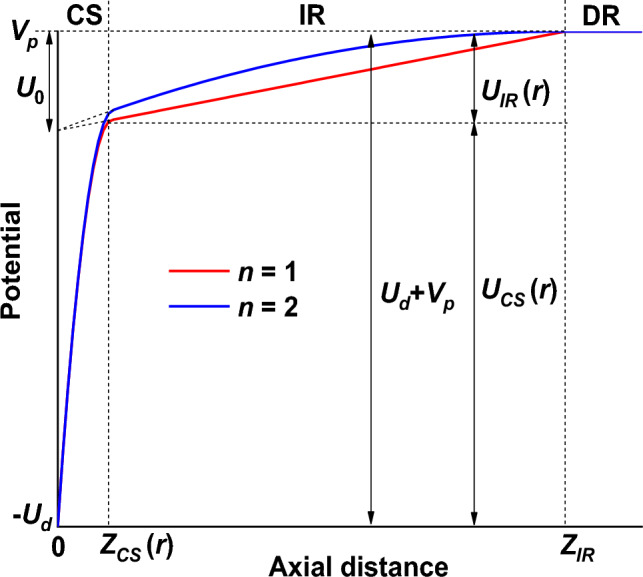
Typical axial distribution of the plasma potential, at any radial position, for the cases *n* = 1 and *n* = 2. The parameters *Z*_*CS*_ and *U*_*IR*_ depend on *r*. *U*_0_ is the intercept at the cathode (*z* = 0) of the voltage drop law in the ionization region (Eq. 2).

Equations (1a) and (1b) assure the continuity with the diffusion region, where the electric field is zero and the potential is *V*_*p*_. However, for a constant electric field in the IR (*n* = 1), only the Eq. (1a) satisfies the continuity condition, the electric field jumping from a non-zero constant value in the IR to zero in the DR. Therefore, the use of a linear electric field (*n* = 2) in the IR seems more appropriate. Moreover, if the voltage drop over the IR is relatively small with respect to the discharge voltage (below about 20%, as it comes out from the IRM^34^), there is not much difference between the two cases (as Fig. 2 shows).

Concerning the axial direction in the cathode sheath, the results of self-consistent numerical simulations reported in the literature show either the electric field, which is quasi-linear^25,33^, or the potential, which can be easily described as parabolic^26,27,30–32^. Laser-induced fluorescence measurements^24^, although at very high pressure (~ 113 Pa), also found linear electric fields in the CS. To be in line with the previous findings, we assume a parabolic dependence of the potential in the axial direction of the cathode sheath, which writes:

3a $${V}_{CS}\left(r,z\right)=a\left(r\right){\left(\frac{{Z}_{CS}\left(r\right)-z}{{Z}_{CS}\left(r\right)}\right)}^{2}+b\left(r\right)\left(\frac{{Z}_{CS}\left(r\right)-z}{{Z}_{CS}\left(r\right)}\right)+c\left(r\right),$$

which results in a linear electric field:

3b $${E}_{CS}\left(r,z\right)=2\frac{a\left(r\right)}{{Z}_{CS}\left(r\right)}\left(\frac{{Z}_{CS}\left(r\right)-z}{{Z}_{CS}\left(r\right)}\right)+\frac{b\left(r\right)}{{Z}_{CS}\left(r\right)}.$$

Equations (3a) and (3b) are valid at each radial position, the parameters *a*, *b* and *c* depending on *r*. At the limit of the cathode sheath, *Z*_*CS*_, both the potential and electric field have to fulfill the continuity conditions, *i.e*., neither the potential nor the electric field exhibit a jump between the CS and IR:

4a $${V}_{CS}\left(r,{Z}_{CS}\right)={V}_{IR}\left({Z}_{CS}\right)$$

4b $${E}_{CS}\left(r,{Z}_{CS}\right)={E}_{IR}\left({Z}_{CS}\right).$$

At the cathode, the potential is fixed by the discharge voltage:

4c $${V}_{CS}\left(r,0\right)=-{U}_{d}.$$

Imposing the boundary conditions (4a)–(4c) to the Eqs. (3a)–(3b) yields the following formulas for *a*(*r*), *b*(*r*) and *c*(*r*):

5a $$a\left(r\right)=-\left({U}_{d}+{V}_{IR}({Z}_{CS})+{E}_{IR}{\left({Z}_{CS}\right)Z}_{CS}(r)\right)$$

5b $$b\left(r\right)={E}_{IR}\left({Z}_{CS}\right){Z}_{CS}\left(r\right)$$

5c $$c\left(r\right)={V}_{IR}\left({Z}_{CS}\right).$$

Replacing the parameters *a*(*r*), *b*(*r*) and *c*(*r*) in the Eqs. (3a)–(3b), one obtains:

6a $${V}_{CS}\left(r,z\right)=-\left({U}_{d}+{V}_{IR}\left({Z}_{CS}\right)+{E}_{IR}{\left({Z}_{CS}\right)Z}_{CS}\left(r\right)\right){\left(\frac{{Z}_{CS}\left(r\right)-z}{{Z}_{CS}\left(r\right)}\right)}^{2}+{E}_{IR}{\left({Z}_{CS}\right)Z}_{CS}\left(r\right)\left(\frac{{Z}_{CS}(r)-z}{{Z}_{CS}(r)}\right)+{V}_{IR}\left({Z}_{CS}\right)$$

6b $${E}_{CS}\left(r,z\right)=-2\frac{{U}_{d}+{V}_{IR}\left({Z}_{CS}\right)+{E}_{IR}{\left({Z}_{CS}\right)Z}_{CS}\left(r\right)}{{Z}_{CS}\left(r\right)}\left(\frac{{Z}_{CS}\left(r\right)-z}{{Z}_{CS}\left(r\right)}\right)+{E}_{IR}\left({Z}_{CS}\right).$$

A typical variation of the plasma potential in the axial direction is schematically shown in Fig. 2 for *n* = 1 and *n* = 2. All other input parameters are identical for the two cases. The curves in Fig. 2 are valid at any radial position, with *Z*_*CS*_ and *U*_*IR*_ depending on *r*.

In the assumption of a non-collisional cathode sheath for ions, which is usually valid in a magnetron discharge working at low pressure (~ 1 Pa or below), and since the ions are not sensitive to the magnetic field, the thickness of the cathode sheath *Z*_*CS*_ is given by the 1D (axial) Child–Langmuir law:

7 $${Z}_{CS}\left(r\right)=\sqrt{\frac{4}{9}{\epsilon }_{0}{\left(\frac{2e}{{m}_{i}}\right)}^\frac{1}{2}\frac{{{U}_{CS}\left(r\right)}^\frac{3}{2}}{{j}_{i}\left(r\right)}},$$

where

8 $${U}_{CS}(r)={U}_{d}+{V}_{p}-{U}_{IR}(r)$$

is the voltage drop over the cathode sheath (Fig. 2). In Eq. (7), *e* is the elementary charge, *m*_*i*_ is the ion mass and *ε*_0_ is the electrical permittivity of vacuum. In this form, the Child–Langmuir law assumes an ion current density to the target *j*_*i*_(*r*) carried by singly-charged positive ions. This assumption holds for most of the magnetron plasmas, except for high power pulses where multiple charged ions are present. Also, the Child–Langmuir theory assumes the continuity of the current density through the dark cathode sheath, whatever the current density is, and this condition holds for any collisionless sheath. Optical emission spectroscopy measurements^23^, performed for different magnetic fields, discharge currents and pressures, found that the mean CS thickness of a DC magnetron discharge scales as the Child–Langmuir law, being typically twice as large. Still, *Z*_*CS*_(*r*) is calculated from the Child–Langmuir law since the optical measurements provided the mean and not the local thickness of the CS and the maximum of the emission intensity is not necessarily identical to the CS limit. The potential in the CS (Eq. 6a) is not described by the Child–Langmuir law (*V* ~ *z*^4/3^) because the latter dependency is not valid the entire sheath, but only close to the cathode. As it was already mentioned, the parabolic dependence (6a) better describes the results reported in the literature.

*Z*_*CS*_(*r*) depends on the radial position via* j*_*i*_(*r*) which can be directly measured using probes embedded into the target^35^. Still, the method itself is a technical challenge. An easier way is the estimation of *j*_*i*_(*r*) from the target erosion profile. The comparison between the experiment and simulations showed that the erosion profile is proportional to the ion energy flux to the target^27,36^. Since the average energy of the ions reaching the target is almost constant over the entire width of the racetrack^31^, the erosion profile can be considered proportional to the ion current density to the target, as confirmed by numerical simulations^5,29^. Consequently, *j*_*i*_(*r*) can be written as:

9 $${j}_{i}(r)=k \chi (r).$$

The constant *k* can be obtained by integrating the ion current density over the entire target, which connects to the discharge current through:

10a $${\int }_{o}^{{R}_{c}}{j}_{i}(r)2\pi rdr=2\pi k{\int }_{o}^{{R}_{c}}\chi (r)rdr=\frac{{I}_{d}}{1+{\gamma }_{eff}},$$

where *R*_*c*_ is the cathode radius and *γ*_*eff*_ is the effective ion induced secondary electron emission yield (ISEY), defined in^37^. For a rectangular target, the integration of the ion current density is calculated as:

10b $$2{\int }_{0}^{w/2}{j}_{i}(x)(L-w+4x)dx=\frac{{I}_{d}}{1+{\gamma }_{eff}},$$

where *L* and *w* are the length and width of the target, respectively. In a magnetron discharge, the magnetic field returns a fraction of the secondary electrons to the cathode, where they might be recaptured by the surface, lowering thus *γ*_*eff*_ with respect to the ISEY measured without a magnetic field^9,38^. If the measured ISEY is below 0.1, the contribution of the secondary electrons to the discharge current can be neglected in the Eqs. (10a) and (10b) since *γ*_*eff*_ is generally less than half of the measured ISEY^9^, and a variation of 5% of the ion current will determine a change of the CS thickness below 4%.

Thus, based on the six input parameters, one can compute *j*_*i*_(*r*), *U*_*IR*_(*r*), and *Z*_*CS*_(*r*) and further obtain the 2D map of the plasma potential in the cathode sheath and ionization region. Equations (2), (7), and (8) show that *U*_*IR*_(*r*) and *Z*_*CS*_(*r*) depend on each other. Their values can be obtained by iterative calculation. Starting with *U*_*IR*_(*r*) = 0, the solution of Eqs. (2), (7), and (8) converges in approximately 5 iterations (faster for *n* = 2 than for *n* = 1).

## Results and discussion

**Figure 3:**
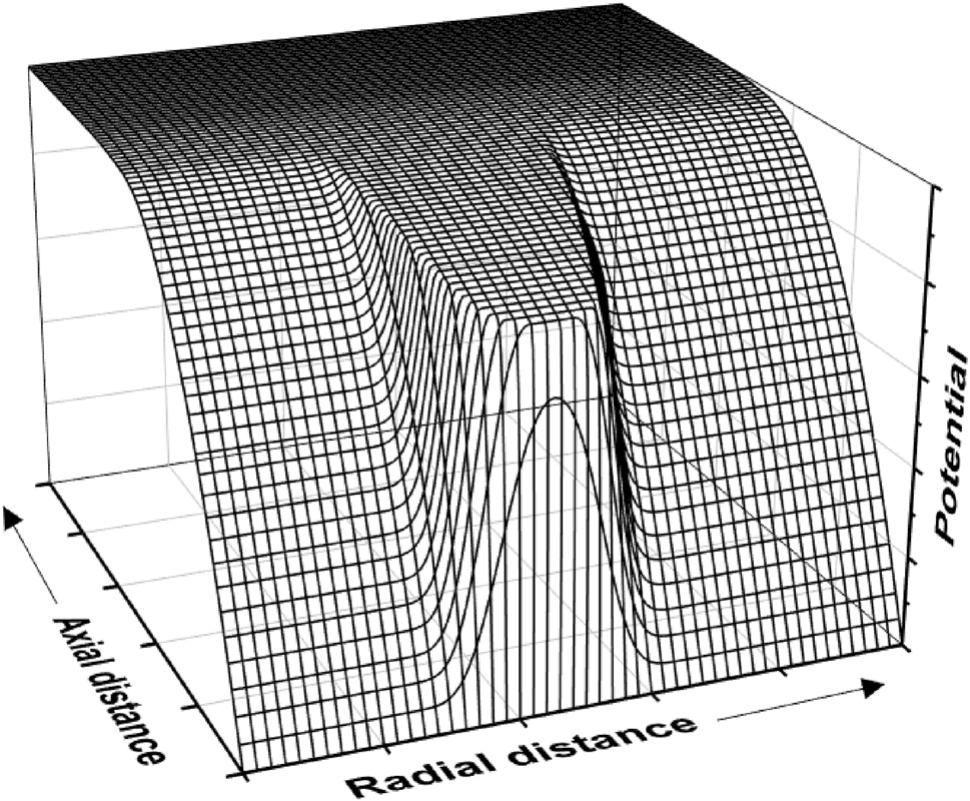
Typical 2D plasma potential distribution resulting from the analytical formulas proposed in this work.

A typical distribution of the plasma potential obtained with the proposed analytical formulas is plotted in Fig. 3. The electric field in the ionization region was taken linear (*n* = 2), and an arbitrarily chosen Gauss function described the target erosion profile. It can be noticed that the analytical formulas capture the general characteristics of the highly inhomogeneous plasma potential reported for DC^25–29^ magnetron discharges. The CS is very narrow in the racetrack region and larger on the sides. Complementarily, the IR extent is much larger in the racetrack region and narrower on the sides. The CS is characterized by an electric field with non-zero components in both *z* and *r* directions. The CS thickness obeys the collisionless Child–Langmuir law (Eq. 7), increasing with the CS voltage drop and decreasing with the discharge current. Concerning other discharge parameters, previous works showed that the CS thickness decreases with the increase of both working pressure^23^ and magnetic field strength^23,25,32^.

In the case of time-dependent discharges, such as HiPIMS, the analytical formulas seem to hold only for particular moments during the pulse (e.g., Fig. 5A′,C′ in^30^). It is challenging to apply them to time-dependent discharges since some input parameters may be difficult to estimate: the erosion profile of the target is the result of a cumulative effect of a time-dependent sputtering process, knowledge of the voltage drop over the IR would require the prior use of other models like, for instance, IRM, etc. Also, in a HiPIMS discharge, the ion current to the cathode contains gas and sputtered material ions, demanding reconsidering the Child–Langmuir law. However, if time-dependent input parameters can be estimated and the conditions discussed below are met, the proposed analytical description can also be used for transitory magnetron discharges.

Several probe measurements^20,21^ and simulations^30,31^ showed a different distribution of the potential in the IR of a HiPIMS discharge, with respect to Fig. 3: the equipotential lines are quasi-circular, the electric field converging towards the racetrack. Thus, for a fixed *z* distance, the potential above the racetrack (at the radial position *r*_*RT*_ in Fig. 1) is more negative than outside the racetrack, contrasting the results shown in Fig. 3 and those of the mentioned PIC simulations^25–30^. In terms of mathematical functions, the potential distribution in the IR is convex in references^25–30^ and concave in references^20,21,30,31^.

Apparently, a concave potential distribution in the IR is characteristic to a HiPIMS discharge, being related to the transitory phenomena that characterize a pulsed discharge. However, recent PIC simulations showed that both convex and concave potential distributions could describe the ionization region of a DC magnetron discharge, depending on the operating pressure^39^. Thus, a concave potential was found to be typical for pressures below approximately 5 mTorr (0.67 Pa), which was also the case in the two HiPIMS experiments (0.54 Pa in^20^ and 0.26 Pa in^21^) and the HiPIMS simulation (0.4 Pa in^31^). In contrast, a convex potential was typical for higher pressures (the results of the other PIC simulations^25–30^ were obtained at a pressure of 0.67 Pa or higher).

The dominant electric charge in the IR gives the difference between concave and convex potential distributions. According to Poisson’s equation, the potential is convex (the second derivative in space is negative) when the total electric charge is positive (excess of ions) and concave when the total electric charge is negative (excess of electrons). Note that the excess of charges does not have to be large; for a plasma density of 10^16^ m^−3^, a difference of only 1% between electron and ion densities already makes the difference between concave and convex potential. The local balance between the gain and loss of electrons determines the dominant charged species in the IR. Electron gain is due to ionizations, while electron loss is related to the transport across the magnetic field. The latter is regulated by the Hall parameter *ω*_*ge*_*τ*_*e*_, where *ω*_*ge*_ is the electron gyrofrequency, and *τ*_*e*_ is the electron momentum transfer time^40^. The higher the Hall parameter, the lower the electron transport across the magnetic field, which means better electron confinement in the IR and, consequently, a possible excess of electrons.

The PIC simulation results reported in^39^ found that the electron balance in the IR favors electron gain at low pressure and loss at higher pressure. At low pressure, the number of electron collisions is low, *τ*_*e*_ is high, and so is the Hall parameter; the electrons are better confined. Even if the ionization rate is low, the outcome is an excess of electrons in the IR and a concave potential. At higher pressure, *τ*_*e*_ and the Hall parameter decrease, facilitating the electron escape from the magnetic trap. Hence, local ion density dominates, and a convex potential develops, even though the ionization rate also increases. The pressure limit between concave and convex potential was found to be approximately 5 mTorr (0.67 Pa) in^39^ for a magnetic field trap characterized by 600–700 G in the racetrack region near the cathode (*B*_*RT*_). All previous discussions followed the Hall parameter, so the pressure limit must change by changing the magnetic field. Therefore, based on the results in^39^, an empirical limit between concave and convex potential can be set to a reduced magnetic field *B*_*RT*_/*p* of approximately 0.1 T/Pa or 130 G/mTorr. Below this limit, the potential is convex, and above it is concave. The limit of *B*_*RT*_/*p* is indicative and is valid for an ionization rate assured by the electron acceleration in a discharge voltage of a few hundred volts (typical for DC discharges). It proved to be reasonable even at lower pressure (*p* = 0.26 Pa) because of the lower magnetic field (*B*_*RT*_ ~ 300 G), the potential in the IR being convex^4^.

In the case of a transitory plasma, the local balance between electron gain and loss is time-dependent. The measurements in^40^ showed that the Hall parameter changes during a HiPIMS discharge, and the ionization rate changes since the discharge voltage can vary by several hundred volts during the pulse^1^. This may lead to convex or concave potential distributions in the IR at different pulse moments, as obtained by simulations^30^.

The Hall parameter also influences the voltage drop over the IR^4,39^ via the working pressure and the magnetic field strength. Thus, *U*_*IR*_ increases when the pressure decreases (Hall parameter increase), reaching about half of the discharge voltage at about 0.67 Pa, as reported in^39^. From self-consistent simulations, *U*_*IR*_ also increases with the magnetic field strength^32^ (i.e., increasing Hall parameter), a result confirmed by several other simulations: lower *U*_*IR*_ values for low magnetic fields (*B*_*RT*_ below 500 G)^26–29^, higher *U*_*IR*_ values for high magnetic fields (*B*_*RT*_ about 800 G)^30,31^. The *U*_*IR*_ increase with the Hall parameter is in line with the electron transport across the magnetic field. A higher Hall parameter means lower electron mobility across the magnetic barrier, which requires a higher electric field (higher *U*_*IR*_) to ensure an equivalent electron flow to the anode (i.e., constant discharge current). In Fig. 3, the voltage drop over the IR was set to 20% of the discharge voltage, in line with the results reported in most of the discussed references.

If some of the input parameters required by the proposed analytical formulas cannot be directly measured, they can be estimated as follows. The erosion profile can be approximated from the spatial distribution of the plasma radiation across the cathode, measured either as globally emitted light, integrated into the axial direction^36^ or at specific wavelengths (of gas atoms or ions) and certain distances from the cathode^41^. Optical measurements are also suitable for transient plasmas. Moreover, numerical simulations showed that the target erosion profile corresponds to the ionization events distribution in the IR volume projected onto the cathode^5^. The limit of the ionization region towards the anode *Z*_*IR*_ can be roughly estimated from optical observations in the axial direction, as the limit of the negative glow^23,41,42^. Also, if the magnetic field configuration is known, *Z*_*IR*_ can be approximated with the extent of the magnetic trap in the axial direction. Both experiments^4,19,22^ and simulations^30,32^ showed, depending on the discharge conditions, that *V*_*p*_ can be slightly positive or negative with respect to the grounded anode. Therefore, if it is not possible to measure *V*_*p*_, it can be neglected with respect to the discharge voltage (|*V*_*p*_|< < *U*_*d*_). The most challenging input parameter is *U*_0_. The voltage drop over the IR can be taken as a fraction of the discharge voltage, according to the results of different experiments^4^, models^34^, or simulations^27,32,39^, selecting the value corresponding to the closest discharge operation condition. If that is not possible, *U*_0_ can be treated as an adjusting parameter.

Besides the results reported in the literature^4,22,24–28,30–33^, the proposed analytical description is directly compared to a self-consistent 2D numerical simulation (PIC). The comparison does not reduce the validity of the analytical formulas to this particular case, but allows a detailed analysis. The PIC simulation was made for a rectangular cathode (40 × 90 mm^2^) with argon gas at 0.67 Pa and a DC discharge voltage of 323 V, using the numerical approach described in^30^, with the same magnetic field configuration. Briefly, the simulation domain (20 × 25.55 mm^2^) was discretized on a regular grid of 201 × 512 nodes. To fulfill the Courant–Friedrichs–Lewy (CFL) stability criterion^43^, the time step was 2.5 × 10^–12^ s. Electrons and singly-charged positive ions (Ar^+^) composed the plasma. The electron pusher routine acts every time step, while the ions move every 10 time steps, known as the sub-cycling technique. Both electrons and ions collide only with neutrals: elastic, excitation, and ionization collisions for electrons; resonant charge transfer and elastic collisions for ions. The collisions were treated using a Monte Carlo technique, taking the collision cross-sections from^44^ for electrons and from^45^ for ions. Poisson’s equation was solved every time step to update the plasma potential and the electric field. The magnetic field configuration was unaffected by the presence of the plasma. At the racetrack location (*x*_*RT*_ ≈ 9.3 mm), where the field lines are parallel to the cathode, the magnetic field strength was *B*_*RT*_ ~ 750 G at the cathode surface. The physical computation time was 15 μs to obtain the global convergence of all plasma parameters (corresponding to the DC steady-state). All technical details of the PIC simulation are given in^30^.

**Figure 4:**
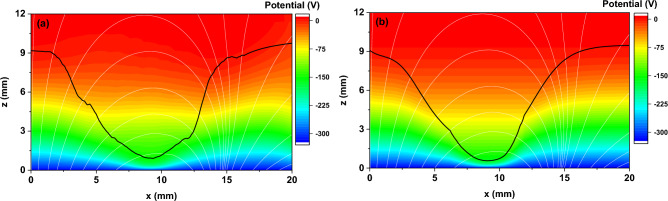
2D maps of the plasma potential obtained from the PIC simulation (**a**) and the proposed analytical description (**b**). The black curves indicate the cathode sheath width, *Z*_*CS*_(*r*), obtained for each separate case. Magnetic field lines are plotted in white.

**Figure 5:**
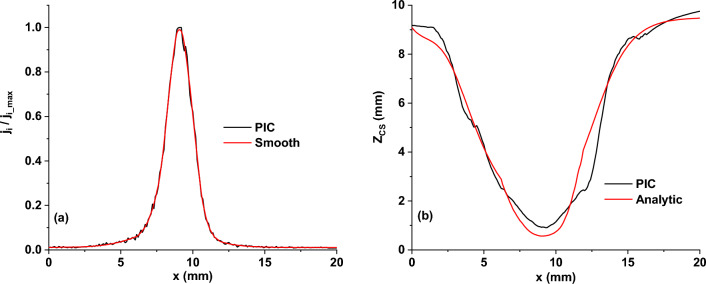
(**a**) Normalized ion current density profile to the cathode resulting from the PIC simulation. (**b**) Comparison of the CS thickness radial profiles obtained from the PIC simulation (black) and the analytical description (red).

**Figure 6:**
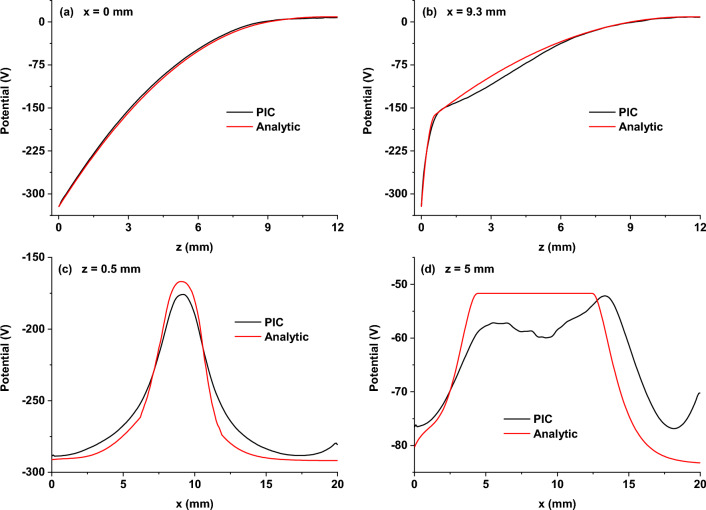
Comparison of 1D profiles extracted from the 2D plasma potential maps in Fig. 4, obtained from the PIC simulation (black) and the analytical description (red), plotted at different transverse and axial positions: (**a**) *x* = 0 mm (on the discharge axis), (**b**) *x* = 9.3 mm (above the racetrack), (**c**) *z* = 0.5 mm (in the CS), (**d**) *z* = 5 mm (in the IR).

Figure 4 compares the two methods' 2D maps of the plasma potential. The input parameters for the proposed analytical formulas were taken from the PIC simulation results: *I*_*d*_ = 25 mA and *V*_*p*_ =  + 9 V. Instead of the experimental target erosion profile, we used the profile of the ion current density to the target directly from PIC (Fig. 5a), smoothing it to reduce the noise. The limit of the ionization region towards the anode (*Z*_*IR*_ = 11.5 mm) and the maximum voltage drop over the IR (*U*_0_ = 190 V) were chosen for the best fit with the PIC simulation. *Z*_*IR*_ was set to be larger than the cathode sheath thickness at any radial position and complied with the negative glow boundary in the PIC simulation. The axial distribution of the potential in the IR was taken parabolic (*n* = 2). The two results in Fig. 4 show a good agreement, but for a thorough analysis, it is more practical to compare 1D profiles of the potential (see Fig. 6). The black curves in Fig. 4 show the extension of the cathode sheath given by each method. In the PIC simulation, the thickness of the CS was set by the appearance of positive/negative fluctuations of the total charge density. In the current approach, the thickness of the CS was calculated using the Eq. (7). A direct comparison of the two CS thickness profiles is shown in Fig. 5b. The profiles match fairly well, the sensitive region being about the racetrack. Precisely at the racetrack location (*x*_*RT*_ ≈ 9.3 mm), the current approach gives *Z*_*CS*_ = 0.6 mm against *Z*_*CS*_ = 0.9 mm for the PIC simulation. The difference might appear from the estimation of *Z*_*CS*_ in PIC. However, this discrepancy does not affect the potential distribution at the limit between the CS and the IR (Fig. 6b).

A selection of 1D profiles extracted from the 2D plasma potential maps in Fig. 4 was plotted in Fig. 6 for different fixed coordinates. The choice of fixed transverse and axial positions was guided by being the most representative discharge regions. Thus, axial 1D profiles of the plasma potential are plotted on the discharge axis (Fig. 6a) and above the racetrack (Fig. 6b). A perfect agreement is observed between the two methods on the discharge axis. The potential is like the one without a magnetic field along the symmetry axis since both electric and magnetic fields are axially oriented and parallel to each other. Above the racetrack, the analytical approach describes well the CS and the anode side of the IR. However, a slight difference from the PIC simulation is noticed in the region 1 < *z* < 6 mm. From the fairly good agreement of the potential distributions, the axial electric fields also match well between the two methods (not shown).

In the transverse direction, 1D profiles of the plasma potential are plotted in the cathode sheath (Fig. 6c) and the ionization region (Fig. 6d). The match between the results of the two methods is less accurate in this direction. However, the importance of the discrepancy in the total electric field is rather small since the transverse electric field is an order of magnitude lower than the axial one. Therefore, a marginal impact is expected on the final results if the 2D map produced by the analytical formulas is further used in subsequent calculations.

## Conclusion

Using a limited number of input parameters, which can be obtained experimentally, the proposed analytical description provides the 2D map of the plasma potential in a steady-state magnetron discharge. The approach affords a mathematical expression of the potential in the axial direction (perpendicular to the target), the variation of the potential in the transverse direction (parallel to the target) being related to the target erosion profile and the 1D collisionless Child–Langmuir law. The second derivative of the plasma potential allows for calculating the total charge density as the source term in Poisson’s equation. The analytical description does not require the knowledge of the magnetic field structure or magnitude to obtain a fair estimate of the plasma potential. In case some input parameters are unavailable from direct measurements, alternative estimation methods were suggested. The most challenging input parameter is the voltage drop over the ionization region.

The thickness of the cathode sheath is sensitive to the ion current density to the target (Eq. 7), especially at very low values of *j*_*i*_. Therefore, the target erosion profile must be cautiously evaluated, particularly in the regions of low erosion (outside the racetrack, where *j*_*i*_ has low values). The sputtered material is often subject to redeposition there^46^, which may mislead the erosion measurement.

The proposed analytical formulas are suitable for steady-state magnetron discharges if the electron loss in the ionization region is higher than the electron gain. This condition is fulfilled when operating with a reduced magnetic field *B*_*RT*_/*p* below approximately 0.1 T/Pa and a discharge voltage of a few hundred volts. The analytical formulas capture all features of the plasma potential of such discharge obtained by self-consistent simulations^25–29^. If the electron loss is lower than the electron gain, the potential in the ionization region might be concave, as found in experimental measurements^20,21^ and simulations^31,39^. For such a case, a mathematical fit formula was proposed in^8^, which has to be linked to the cathode sheath equations.

The analytical approach described in this work gives a fast way to approximate the 2D map of the plasma potential in the very sensitive regions of the magnetron discharge, namely the cathode sheath and the ionization region, providing input for experimental data interpretation or subsequent calculations.

## Data Availability

The datasets used and/or analyzed during the current study available from the corresponding author on reasonable request.
